# Antimicrobial Resistance, Virulence, and Genetic Lineages of Staphylococci from Horses Destined for Human Consumption: High Detection of *S. aureus* Isolates of Lineage ST1640 and Those Carrying the *lukPQ* Gene

**DOI:** 10.3390/ani9110900

**Published:** 2019-11-01

**Authors:** Olouwafemi Mistourath Mama, Paula Gómez, Laura Ruiz-Ripa, Elena Gómez-Sanz, Myriam Zarazaga, Carmen Torres

**Affiliations:** 1 Departamento de Agricultura y Alimentación, Universidad de La Rioja, 26006 Logroño, Spain; femimama92@hotmail.com (O.M.M.); paula_gv83@hotmail.com (P.G.); laura_ruiz_10@hotmail.com (L.R.-R.); myriam.zarazaga@unirioja.es (M.Z.); 2 Laboratory of Food Microbiology, Institute of Food, Nutrition and Health, ETH Zurich, 8092 Zurich, Switzerland; 3 Área de Microbiología Molecular, Centro de Investigación Biomédica de La Rioja (CIBIR), 26006 Logroño, Spain

**Keywords:** healthy horses, staphylococci, MSSA, ST1640, *lukPQ*

## Abstract

**Simple Summary:**

Staphylococci are opportunistic pathogens which colonize humans and animals. Zoonotic transfer of staphylococcal species between domestic animals and humans is common and can occur through direct contact, the environment, and animal-derived food processing, implying a risk of the spread of antimicrobial resistance mechanisms and virulence factors into different ecosystems. Our work aimed at studying the diversity of staphylococcal species in nasal and faecal samples of healthy horses intended for human consumption and their resistance and virulence determinants. Staphylococci were detected in 90% and 66% of nasal and faecal samples tested, respectively. Eight staphylococcal species were detected, with the most prevalent ones being *Staphylococcus aureus* (all isolates were methicillin-susceptible), *Staphylococcus delphini*, and *Staphylococcus sciuri*. The predominant *S. aureus* lineage, ST1640, is associated with horses for the first time in this study. *S. aureus* isolates, except those of lineage ST1640, produced equid-adapted leukocidin (LukPQ) and blocker of equine complement system activation (eqSCIN). The toxic shock syndrome toxin-encoding gene was also detected in some *S. aureus* isolates. Multidrug resistance was observed among *S. sciuri* isolates, but not among *S. aureus.* Measures of hygiene and control should be implemented during horse slaughter and meat processing.

**Abstract:**

This work aimed to determine the frequency and diversity of *Staphylococcus* species carriage in horses intended for human consumption, as well as their resistance and virulence determinants. Eighty samples (30 nasal; 50 faecal) were recovered from 73 healthy horses in a Spanish slaughterhouse. The samples were cultured for staphylococci and methicillin-resistant staphylococci (MRS) recovery. The phenotype/genotype of antimicrobial resistance was analysed for all isolates. The *spa*-type and sequence-type (ST) were determined in *Staphylococcus aureus* strains; moreover, the presence of virulence and host-adaptation genes (*tst*, *eta, etb, pvl*, *lukPQ*, *scn-eq,* and *scn*) was studied by PCR. *Staphylococcus* species were detected in 27/30 (90%) and 33/50 (66%) of nasal and faecal samples, respectively. Ninety isolates belonging to eight species were recovered, with predominance of *S. aureus* (*n* = 34), *Staphylococcus delphini* (*n* = 19), and *Staphylococcus sciuri* (*n* = 19). *S. aureus* strains were all methicillin-susceptible (MSSA), 28/34 were susceptible to all the antibiotics tested, and the remaining six showed resistance to (gene-detected) streptomycin (*ant* (6)-*Ia*), penicillin (*blaZ*), and trimetroprim/sulphametoxazole (SXT) (*dfrA*, *dfrG*). The lineage ST1640/t2559 was predominant (*n* = 21). The genes *lukPQ* and *scn-eq* were present in all but the ST1640 isolates. Three *S. sciuri* isolates were multidrug-resistant. Healthy horses in Spain seem to be a reservoir for virulent MSSA and the lineage ST1640, although the presence of the latter in horses is described for the first time in this study. Moreover, the equine-adapted leukocidin gene *lukPQ* is frequent among *S. aureus* strains. A large variety of staphylococcal species with low antibiotic resistance rate were also observed.

## 1. Introduction

Staphylococci are commensal bacteria that generally colonize nares, skin, and mucous membranes of humans and of wild and domestic animals, although some species are opportunistic pathogens [1,2,3,4,5,6]. Horses have been described as carriers of staphylococcal species and methicillin-resistant staphylococci (MRS) [7,8,9,10]. Coagulase-positive staphylococci (CoPS) such as *Staphylococcus aureus*, *Staphylococcus intermedius*, *Staphylococcus delphini,* and *Staphylococcus pseudintermedius* are frequently reported as colonizers or infectious agents in horses [8,9,10,11,12]. Coagulase-negative staphylococci (CoNS) have been described as causative agents of mastitis, wound infections, and skin abscesses in various animals, including horses [13].

Staphylococcal infections are a major issue in both human and veterinary medicine, and their role in severe diseases has increased with the acquisition of antimicrobial resistance mechanisms [11,13]. Moreover, *S. aureus* has a large variety of virulence factors, such as staphylococcal enterotoxins, toxic shock syndrome toxin (TSST-1), or leukocidins, among others [14]. Leukocidins are a family of bicomponent pore-forming toxins involved in *S. aureus* pathogenicity [15]. To date, six leukocidins have been identified, including Panton Valentine leukocidin (*lukF/lukS-PV*), LukMF’, and the novel equid-adapted leukocidin LukPQ, which are related to phage-encoded genes mainly found in humans, ruminants, and equines, respectively [15,16]. LukPQ, encoded by the 45-kb prophage *φ*Saeq1, was found to be strongly associated with *S. aureus* from horses and donkeys. This leukocidin preferentially destroys neutrophils with higher efficiency than its closest fellow, LukED [15]. It was recently revealed that the prophage *φ*Saeq1 also encodes a novel variant of staphylococcal complement inhibitor SCIN-A (termed eqSCIN, encoded by *scn-eq*) which shares 57.8% amino acid identity with SCIN-A (encoded by *scn*) from human *S. aureus* [17]. eqSCIN is a potent blocker of equine complement system activation, which plays an important role in *S. aureus* host adaptation. Whereas SCIN-A isolates exclusively inhibit human complement, eqSCIN represents the first animal-adapted SCIN variant that functions in a broader range of hosts (horses, humans, and pigs) [17].

The presence of staphylococcal species in horses is of public health concern since the potential transfer of *Staphylococcus* spp. and their resistance and virulence genes between healthy humans and domestic animals has been evidenced [18,19,20]. Direct contact may be a way of transmission, but other vehicles, such as the environment and food, should be taken into consideration. In Spain, horse meat is used for human meat consumption; hence, it is important to determine the diversity of staphylococcal species colonizing the mentioned animal species. In that context, this work aimed to identify the different species of staphylococci present in nares and faeces of healthy horses destined to human consumption, as well as the antimicrobial resistance phenotype and genotype of the recovered isolates, and the virulence traits for *S. aureus* species.

## 2. Material and Methods

### 2.1. Sample Recovery

A total of 80 samples (nasal: *n* = 30 and faecal: *n* = 50) were recovered with sterile swabs from 73 healthy horses intended for human consumption and kept in Amies transport medium (Copan, Murrieta/USA). Seven animals were tested for both types of samples. Animals came from 19 Spanish regions before they were transported to a slaughterhouse located in Northern Spain, where samples were taken in February 2012.

### 2.2. Staphylococcus spp. Isolation, Identification, and DNA Extraction

The samples were first inoculated in Brain Heart Infusion (BHI, supplemented with NaCl 6.5%) broth (Conda, Madrid/Spain) and incubated at 37 °C for 24 h. After growth, the bacterial culture was distributed on plates of mannitol–salt–agar (Conda, Madrid/Spain) and oxacillin resistance screening agar base (Oxoid, Hampshire/England) for staphylococci and MRS recovery, respectively. Up to four colonies/plate with staphylococcal morphology were isolated and subjected to the DNase agar test (Conda, Madrid/Spain). Identification was performed by PCR (for CoPS isolates) [21] and by matrix-assisted laser desorption/ionization time of flight (MALDI-TOF) mass spectrometry (Bruker, MA, USA) (for all the staphylococci).

The DNA extraction was performed as follows: one colony was resuspended in 45 µL of milli-Q water and 5 µL of lysostaphin (1 mg/mL). The suspension was warmed in a water bath at 37 °C during 10 min. Then 45 µL of milli-Q water, 150 µL of Tris (0.1 M, pH 8.5), and 5 µL of proteinase K (2 mg/mL) were added to the suspension before it was heated again in a water bath at 60 °C for 10 min and at 100 °C for 5 min. Finally, centrifugation was performed at 12,000 rpm for 3 min and the supernatant was kept for further experiences.

### 2.3. Antimicrobial Susceptibility and Resistance Genes

Susceptibility testing to penicillin, cefoxitin, gentamicin, tobramycin, tetracycline, erythromycin, clindamycin, chloramphenicol, ciprofloxacin, linezolid, and trimethoprim/sulfamethoxazole (SXT) was performed by disk-diffusion method according to the Clinical Laboratory Standards Institute recommendations [22]. Susceptibility to streptomycin was also tested (CASFM 2018). The presence of the following antimicrobial resistance genes was determined by PCR, in accordance with the identified resistance phenotypes: beta-lactams (*mecA*, *blaZ*), tetracycline (*tet*(K), *tet*(L), and *tet*(M)), macrolides-lincosamides (*erm*(A), *erm*(B), *erm*(C), *erm*(T), *msr*(A), *lnu*(A), *lnu*(B), and *vgaA*), streptomycin (*str* and *ant*(6)-Ia), chloramphenicol (*fexA* and *fexB*), and SXT (*dfrA*, *dfrD*, *dfrG* and *dfrK*) [23,24,25,26,27].

### 2.4. Molecular Typing

For the *S. aureus* isolates, *spa*-typing was performed as previously described [28]. Multilocus sequence typing (MLST) was determined for representative isolates (one isolate of each *spa*-type, except for the *spa*-type t2559 for which three associated isolates were chosen). For this purpose, PCR and sequencing of seven housekeeping genes (www.pubmlst.org) were performed to define the sequence type (ST) and the clonal complex (CC). Additionally, detection of *agr* allotypes was carried out by two multiplex PCRs in all isolates [29]. For *S. delphini* isolates, a PCR with specific primers was performed to classify them in two groups (A or B) as previously described [30].

### 2.5. Virulence Genes

For *S. aureus* isolates, the presence of the genes encoding the toxic shock syndrome toxin (*tst*) and exfoliative toxin A (*eta*) and B (*etb*) was studied by PCR [26]. In addition, the genes encoding the leukocidins of Panton-Valentine (*lukF*/*lukS*-PV) and LukPQ were studied by PCR and sequencing [15,26]. The presence of *scn* (gene for SCIN-A) [31] and *scn-eq* (gene for eqSCIN) was analysed by PCR and sequencing. The *scn-eq* PCR was performed using a pair of primers designed in this study (eqSCN-F: TGCTGCTTTTGCTTTGTATCC and eqSCN-R: TGCAGGAGTTTTAGTTGCAGTTTT) and the following conditions: 94 °C for 3 min, followed by 30 cycles of 1 min at 94 °C, 2 min at 61.5 °C, and 3 min at 72 °C, with a final extension at 72 °C for 10 min.

## 3. Results

*Staphylococcus* isolates were detected in 27/30 (90%) of nasal samples and in 33/50 (66%) of faecal samples. The seven animals tested for both types of samples were positive for staphylococcal species in both cases.

A total of 90 isolates of eight species were detected in the positive nasal/faecal samples: *S. aureus* (*n* = 34), *S. delphini* (*n* = 19), *Staphylococcus sciuri* (*n* = 19), *Staphylococcus simulans* (*n* = 4), *Staphylococcus fleurettii* (*n* = 2), *Staphylococcus lentus* (*n* = 2), *Staphylococcus saprophyticus* (*n* = 2), *Staphylococcus xylosus* (*n* = 2), *Staphylococcus haemolyticus* (*n* = 2), *Staphylococcus schleiferi* (*n* = 2), *Staphylococcus vitulinus* (*n* = 1), and *Staphylococcus hyicus* (*n* = 1) (Table 1 and Table 2). Twenty-four samples harboured at least two isolates of either distinct species, distinct antibiotic resistance phenotypes or different *spa*-types.

### 3.1. S. aureus Isolates: Molecular Characteristics, Antimicrobial Resistance, and Virulence Determinants

*S. aureus* was detected in 17/30 (56.6%) and 16/50 (32%) of the nasal and faecal samples, respectively. The 34 isolates recovered (nasal origin: *n* = 18; faecal origin: *n* = 16) were ascribed to five *spa*-types (t2559, t3269, t127, t1294 and t549), four STs (ST1640, ST1, ST816 and ST1660), and four *agr-*types (I, II, III and IV) (Table 1). One isolate per sample was detected except for one nasal sample which harboured two *S. aureus* isolates of different *spa*-types (t2559 and t1294). Most of the isolates were susceptible to all the antimicrobials tested (*n* = 28; 82.4%), while the remaining six showed the following resistance phenotypes (number of isolates; genes detected): streptomycin (3; *ant*(6)-*Ia*), penicillin+streptomycin (1; *blaZ*, *ant*(6)-*Ia*, *str*), and penicillin + SXT (2; *blaZ*, *dfrA*, *dfrG*). In addition, three nasal isolates (all ST816) hosted the gene *tst*. Moreover, all isolates but those of the lineage ST1640 harboured the genes *lukPQ* and *scn-eq*. The isolates of lineage ST1640 were collected from animals which came from six regions, most of them of Southern Spain. 

The major *S. aureus* lineage in horses found in this study was the ST1640 associated to nine and 12 strains of nasal and faecal origins, respectively. In addition, the antimicrobial resistance rates, phenotypes and genotypes are similar in isolates of both origins (Table 1). Concerning the virulence genes, the isolates of the lineage ST1640 were the only ones which lacked the LukPQ determinants. None of the isolates of this study harboured the *scn* gene, a marker of the human immune evasion cluster (IEC).

### 3.2. Non-aureus Staphylococcus Species: Molecular Characteristics, and Antimicrobial Resistance

Among the other species identified, one belonged to *Staphylococcus intermedius* group (SIG), *S. delphini,* and was present in 11 (36.6%) of the nasal samples and in eight (16%) of the faecal samples. Nineteen *S. delphini* isolates were detected in total (type B: *n* = 17; type A: *n* = 2). All isolates were susceptible to the antimicrobials tested (Table 2).

The 37 remaining isolates belonged to coagulase-negative staphylococci (CoNS) group. CoNS were present in 16 (53.3%) of the nasal samples and 15 (30%) of the faecal samples. One isolate per sample was recovered except for five samples which harboured two or three isolates of distinct species. Resistance to at least one antimicrobial agent was detected in 52.6% and 44.4% of the nasal and faecal isolates, respectively. Seventy percent of the nasal resistant isolates and 100% of the faecal resistant isolates belonged to the predominant species *S. sciuri*. The other species with resistant isolates were *S. lentus* and *S. xylosus*. Moreover, three *S. sciuri* isolates showed a multidrug resistance (MDR) phenotype, meaning that they were resistant to one agent in three or more antimicrobial categories (Table 2). Globally, the following rates, phenotypes, and genotypes of antimicrobial resistance were reported among CoNS isolated from horses (detection rate; resistance genes detected): penicillin and cefoxitin (37.8%; *mecA*), erythromycin (5.4%; *erm* (A), *erm* (B), *erm* (C) or *msr* (A)), clindamycin (10.8%; *lnu* (A)), tetracycline (10.8%; *tet* (K) and *tet* (L)), streptomycin (13.5%; *str*), and chloramphenicol (2.7%; *fexA*).

### 3.3. Comparison of Nasal and Faecal Samples of Seven Healthy Horses

Seven animals could be tested for both nasal and faecal samples. The results are displayed in Table 3. All the animals that carried *S. aureus* in their nostrils also had this microorganism in their faeces (*n* = 5). In 3/5 cases, the *S. aureus* isolates belonged to the same genetic lineage ST1640. More than one staphylococcal species was detected in five of seven nasal samples, while faecal samples predominantly carried a single staphylococcal species (*S. aureus* in 5/7 cases). The lineage ST1640 was predominant in both nasal and faecal samples.

## 4. Discussion

High *S. aureus* occurrence has been detected among both nasal and faecal samples of healthy horses destined for human consumption (56.6% and 32%, respectively). According to previous works on healthy horses from various farms in Germany and Denmark, the occurrence of *S. aureus* in nasal samples was much lower (6.7% and 13.5%, respectively) [32,33]. Alternatively, a recent Italian study showed that the prevalence of methicillin resistant *S. aureus* (MRSA) in horses tested in slaughterhouses (7%) was significantly higher than those tested on farms and racecourses [34]. In our study, however, no MRSA was detected among the population tested. Islam and collaborators observed that 63.3% of the *S. aureus* strains recovered were MSSA strains, mostly assigned to ST1/t127 and ST1660/t549 [33]. However, the lineages ST1, ST1660, and ST133 are also frequent among MSSA from horses [32,33,35]. Our strains were mostly associated to ST1640/t2559 (*n* = 21), although ST1 (*n* = 8), ST1660 (*n* = 1), and ST133 were also detected. To our knowledge, the lineage ST1640/t2559 is here detected for the first time among horse samples. Nonetheless, the *spa*-type t2559 was previously found (associated to CC5/CC30) in the nostrils of patients of general practitioners with no sign of infections in the Netherlands [36]. The lack of the *scn* gene suggests that none of the strains were of human origin.

Interestingly, all strains but those of the lineage ST1640/t2559 harboured the equine-adapted leukocidin determinant *lukPQ* (prevalence of 38%) and the *scn-eq* gene. These findings suggest that the ST1640 might have jumped recently from another source to the equine environment. On the other hand, an international equid collection study reported *lukPQ* values ranging from 0% to 50%, indicating either (1) the absence of these genes may also be a common feature among horse isolates or, again, (2) a reflection of an early phase of those isolates in the adaptation to this host [15]. Otherwise, it was revealed that *lukPQ* and *scn-eq*, both encoded by the prophage *φ*Saeq1, are prone to occur together and were associated with the clonal complexes CC1, CC133, CC1660, CC350, and CC522 [15,17]. These findings are confirmed by our results (*lukPQ* and *scn-eq* genes associated with CC1, CC133, CC1660, and ST816). The phage-encoded leukocidin LukPQ displays a high toxicity towards equine neutrophils, while the eqSCIN blocks complement activity in equine serum, which implies an important role in the evasion of *S. aureus* of the equid host defence mechanism [15,17]. Moreover, LukPQ has a broad host range as at high concentrations it is capable of lysing bovine and to some extent human neutrophils. Its transmission to human *S. aureus* strains could enhance its pathogenicity. The toxic shock syndrome gene *tst* was detected in strains of ST816, even though *tst* is generally observed among small ruminant isolates [4,35,37]. The presence of these virulence factors in healthy horses destined for human consumption might be of concern for food security and public health since it can spread through handling and processing. 

Regarding the antimicrobial resistance, a low prevalence of resistant strains among *S. aureus* isolates was observed (17.6%, *n* = 4). They showed resistance to penicillin, streptomycin, and SXT, which are antibiotics frequently used in veterinary medicine [38]. 

Other staphylococcal species were identified from the horses studied, with predominance of *S. delphini* and *S. sciuri*. *S. delphini* is described as a colonizer of a wide variety of animal species (Equidae, Mustelidae, dolphins, pigeons, cinerous vulture, among others) [9,39,40,41]. Here, the *S. delphini* group B revealed a predominance in horses. A similar trend was observed by Stull and collaborators in Canada [9], as well as in wild birds in Spain [40]. The high susceptibility to the antibiotics observed among our strains was in accordance with previous results in donkeys and might be due to a lower selective pressure exerted on these animal species [9,41]. Unfortunately, data on antimicrobial therapy or exposure level of these animals were not available.

*S. sciuri* was the predominant species with resistance to methicillin, as previously reported among equine staphylococcal isolates [42]. This species hosts a native *mecA* homologue (*mecA1*) estimated to be the origin of the *mecA* gene for MRS [42]. Three of our strains showed an MDR phenotype. Those resistance genes could be disseminated among horses and humans through contact and derived food manipulation, which would be a risk for animals and human health. In fact, CoNS and methicillin resistant CoNS of the species detected in this study (*S. epidermidis*, *S. haemolyicus*, *S. sciuri*, *S. xylosus*, or *S vitulinus* among others) are frequently isolated from healthy and infected horses and, sometimes, among equine personnel [13,32,42,43]. 

On the other hand, comparison of staphylococcal carriage between the nostrils and the faeces among several animals in this study indicate a higher carriage rate in nasal samples. These results are in agreement with former data, which describe human and animal skin and mucosa, especially the nares, as the most frequent carriage site for staphylococci [3,44]. Remarkably, our results indicate that the gut and nasal microbiota of these animals is similar when referring to staphylococcal species. 

## 5. Conclusions

This study provides data on the staphylococcal carriage of healthy horses. A high prevalence of MSSA, mostly susceptible to the antibiotics tested but carrying important virulence genes (*lukPQ*, *scn-eq,* and *tst*), is highlighted. The detection and predominance of the *S. aureus* lineage ST1640 in horses is noteworthy, as it represents its first description in horses. Furthermore, a high diversity of species among non-*S. aureus* isolates was observed, including CoPS and MRCoNS. Due to current evidence on the influence of animal-derived food in the dissemination of staphylococci and their resistance and virulence genes, strict measures of hygiene and control must be taken for horses at slaughter and for meat processing.

## Figures and Tables

**Table 1:** Characteristics of 34 *Staphylococcus aureus* isolates recovered from nasal and faecal samples of horses at a slaughterhouse.

Sample Source	Number of Strains	ST/CC ^a^ (Number of Strains)	*Spa*-type (Number of Strains)	*Agr-Type*	Antimicrobial Resistance		Virulence Genes (Number of Strains)
					Phenotype ^b^ (Number of Strains)	Genotype (Number of Strains)	
Nasal	18	ST1640 (9)	t2559 (9)	IV	STR (1)	*ant(6)-Ia*	-
					SUSCEPTIBLE (8)	*-*	-
		ST1/CC1 (5)	t3269 (3)	III	SUSCEPTIBLE (3)	-	*lukPQ* (3), *scn-eq* (3)
			t127 (2)	III	PEN, SXT (1)	*blaZ*, *dfrG*	*lukPQ, scn-eq*
					SUSCEPTIBLE (1)	-	*lukPQ, scn-eq*
		ST816/CC479 (3)	t1294 (3)	II	SUSCEPTIBLE (3)	-	*tst* (3), *lukPQ* (3), *scn-eq* (3)
		ST1660/CC9 (1)	t549 (1)	II	PEN, STR	*blaZ, ant(6)-Ia, str*	*lukPQ, scn-eq*
Faecal	16	ST1640 (12)	t2559 (12)	IV	STR (1)	-	-
					SUSCEPTIBLE (11)	-	-
		ST1/CC1 (3)	t127 (2)	III	PEN, SXT (1)	*blaZ*, *dfrA*, *dfrG*	*lukPQ, scn-eq*
			t386 (1)	III	SUSCEPTIBLE (1)	-	*lukPQ, scn-eq*
					STR	-	*lukPQ, scn-eq*
		ST133/CC133 (1)	t2420 (1)	I	SUSCEPTIBLE	-	*lukPQ, scn-eq*

^a^ The multilocus sequence typing (MLST) was performed for one isolate of each *spa*-type, and for three isolates of *spa*-type t2559; ^b^ PEN: penicillin; STR: streptomycin; SXT: trimethoprim/sulphametoxazole.

**Table 2:** Characteristics of 56 non-*aureus* staphylococcal isolates recovered from nasal and faecal samples of horses at the slaughterhouse.

Sample Source	(Number of Strains)	Species (Number of Strains)	Antimicrobial Resistance	
			Phenotype ^c^ (Number of Strains)	Genotype (Number of Strains)
Nasal	30	*S. delphini*^a^ (11)	SUSCEPTIBLE (11)	*-*
		*S. sciuri* (10)	STR (1)	*str (1)*
			PEN, FOX (2)	*mecA* (2)
			PEN, FOX, TET (2)	*mecA* (2), *tet*(K) (2), *tet*(L) (2)
			PEN, FOX, CLI (1)	*mecA*, *lnu*(A) (1)
			PEN, FOX, STR, TET^e^ (1)	*mecA*, *str*, *tet*(K), *tet*(L) (1)
			SUSCEPTIBLE (3)	-
		*S. fleurettii* (2)	SUSCEPTIBLE (2)	-
		*S. lentus* (2)	ERY, CLI ^d^ (1)	*erm*(A), *erm*(B), *msr*(A) (1)
			PEN, FOX, STR (1)	*mecA*, *str* (1)
		*S. saprophyticus* (2)	SUSCEPTIBLE (2)	-
		*S. xylosus* (2)	CLI (1)	-
			SUSCEPTIBLE (1)	-
		*S. haemolyticus* (1)	SUSCEPTIBLE (1)	-
Faecal	26	*S*. *delphini*^b^ (8) *S. sciuri* (9)	SUSCEPTIBLE (8) PEN, FOX (6)	- *mecA* (6)
			PEN, FOX, STR, TET ^e^ (1)	*mecA*, *str*, *tet*(K), *tet*(L) (1)
			ERY, CLI ^d^, STR, CHL ^e^ (1)	*erm*(C), *str*, *fexA* (1)
			SUSCEPTIBLE (1)	-
		*S. simulans* (4)	SUSCEPTIBLE (4)	-
		*S. schleiferi* (2)	SUSCEPTIBLE (2)	-
		*S. haemolyticus* (1)	SUSCEPTIBLE (1)	-
		*S. vitulinus* (1)	SUSCEPTIBLE (1)	-
		*S. hyicus* (1)	SUSCEPTIBLE (1)	-

^a^ type B: *n* = 10; type A: *n* = 1; ^b^ type B: *n* = 7; type A: *n* = 1; ^c^ PEN: penicillin, FOX: cefoxitin, ERY: erythromycin, CLI: clindamycin, TET: tetracycline, STR: streptomycin, CHL: chloramphenicol; ^d^ inducible resistance phenotype. ^e^ multidrug resistance phenotype.

**Table 3:** Comparison of staphylococci recovered from nasal and faecal samples from seven healthy horses.

Animal	Nasal Samples			Faecal Samples		
Animal Code	Species Detected (Number of Strains)	Type A/B or *spa*-type /ST	Antimicrobial Resistance Phenotype ^a^	Species Detected (Number of Strains)	Type A/B or *spa*-Type/ST	Antimicrobial Resistance Phenotype ^a^
1	*S. delphini* (1)	Type B	SUSCEPTIBLE	*S. simulans* (1)	-	SUSCEPTIBLE
	*S. haemolyticus* (1)	-	SUSCEPTIBLE			
25	*S. aureus* (1)	t2559/ST1640	SUSCEPTIBLE	*S. aureus* (1)	t2420/ST133	SUSCEPTIBLE
26	*S. aureus* (1)	t2559/ST1640	SUSCEPTIBLE	*S. aureus* (1)	t2559/ST1640	SUSCEPTIBLE
	*S. sciuri* (1)	-	PEN, FOX, STR, TET			
27	*S. aureus* (1)	t549/ST1660	PEN, STR	*S. aureus* (1)	t127/ST1	PEN, SXT
	*S. delphini* (1)	Type A	SUSCEPTIBLE	*S. delphini* (1)	Type B	SUSCEPTIBLE
28	*S. aureus* (1)	t2559/ST1640	SUSCEPTIBLE	-	-	-
29	*S. aureus* (1)	t2559/ST1640	SUSCEPTIBLE	*S. aureus* (1)	t2559/ST1640	SUSCEPTIBLE
	*lentus* (1)	-	PEN, FOX, STR			
30	*S. aureus* (1)	t2559/ST1640	SUSCEPTIBLE	*S. aureus* (1)	t2559/ST1640	SUSCEPTIBLE
	*S. sciuri* (1)	-	SUSCEPTIBLE			

^a^ PEN: penicillin; FOX: cefoxitin; TET: tetracycline; STR: streptomycin; SXT: trimetroprim-sulphametoxazole.

## References

[1] von Eiff C., Peters G., Heilmann C. (2002). Review Pathogenesis of infections due to coagulase- negative staphylococci. Lancet Infect. Dis..

[2] Walther B., Tedin K., Lübke-Becker A. (2017). Multidrug-resistant opportunistic pathogens challenging veterinary infection control. Vet. Microbiol..

[3] Kluytmans J.A.J.W. (2010). Methicillin-resistant *Staphylococcus aureus* in food products: Cause for concern or case for complacency?. Clin. Microbiol. Infect..

[4] Mama O.M., Gómez-Sanz E., Ruiz-Ripa L., Gómez P., Torres C. (2019). Diversity of staphylococcal species in food producing animals in Spain, with detection of PVL-positive MRSA ST8 (USA300). Vet. Microbiol..

[5] Mama O.M., Ruiz-Ripa L., Fernández-Fernández R., González-Barrio D., Ruiz-Fons J.F., Torres C. (2019). High frequency of coagulase-positive staphylococci carriage in healthy wild boar with detection of MRSA of lineage ST398-t011. FEMS Microbiol. Lett..

[6] Mama O.M., Ruiz-Ripa L., Lozano C., González-Barrio D., Ruiz-Fons J.F., Torres C. (2019). High diversity of coagulase negative staphylococci species in wild boars, with low antimicrobial resistance rates but detection of relevant resistance genes. Comp. Immunol. Microbiol. Infect. Dis..

[7] Aslantas Ö., Türkyilmaz S., Yilmaz M.A., Erdem Z., Demir C. (2012). Isolation and molecular characterization of Methicillin-Resistant Staphylococci from horses, personnel and environmental sites at an equine hospital in Turkey. J. Vet. Med. Sci..

[8] Burton S., Reid-Smith R., McClure J.T., Weese J.S. (2008). *Staphylococcus aureus* colonization in healthy horses in Atlantic Canada. Can. Vet. J..

[9] Stull J.W., Slavić D., Rousseau J., Scott Weese J. (2014). *Staphylococcus delphini* and Methicillin-Resistant *S. pseudintermedius* in horses, Canada. Emerg. Infect. Dis..

[10] Tirosh-Levy S., Steinman A., Carmeli Y., Klement E., Navon-Venezia S. (2015). Prevalence and risk factors for colonization with methicillin resistant *Staphylococcus aureus* and other Staphylococci species in hospitalized and farm horses in Israel. Prev. Vet. Med..

[11] Oguttu J.W., Qekwana D.N., Odoi A. (2017). An Exploratory Descriptive study of antimicrobial resistance patterns of *Staphylococcus* Spp. Isolated from horses presented at a veterinary teaching hospital. BMC Vet. Res..

[12] Gómez-Sanz E., Simón C., Ortega C., Gómez P., Lozano C., Zarazaga M., Torres C. (2014). First detection of methicillin-resistant *Staphylococcus aureus* ST398 and *Staphylococcus pseudintermedius* ST68 from hospitalized equines in Spain. Zoonoses Public Health.

[13] Moodley A., Guardabassi L. (2009). Clonal spread of methicillin-resistant coagulase-negative staphylococci among horses, personnel and environmental sites at equine facilities. Vet. Microbiol..

[14] Jans C., Merz A., Johler S., Younan M., Tanner S.A., Kaindi D.W.M., Wangoh J., Bonfoh B., Meile L., Tasara T. (2017). East and West African milk products are reservoirs for human and livestock-associated *Staphylococcus aureus*. Food Microbiol..

[15] Koop G., Vrieling M., Storisteanu D.M.L., Lok L.S.C., Monie T., Van Wigcheren G., Raisen C., Ba X., Gleadall N., Hadjirin N. (2017). Identification of LukPQ, a novel, equid-adapted leukocidin of Staphylococcus aureus. Sci. Rep..

[16] McCarthy A.J., Lindsay J.A. (2013). *Staphylococcus aureus* innate immune evasion is lineage-specific: A bioinfomatics study. Infect. Genet. Evol..

[17] De Jong N.W.M., Vrieling M., Garcia B.L., Koop G., Brettmann M., Aerts P.C., Ruyken M., Van Strijp J.A.G., Holmes M., Harrison E.M. (2018). Identification of a staphylococcal complement inhibitor with broad host specificity in equid *Staphylococcus aureus* strains. J. Biol. Chem..

[18] Gómez-Sanz E., Torres C., Lozano C., Zarazaga M. (2013). High diversity of *Staphylococcus aureus* and *Staphylococcus pseudintermedius* lineages and toxigenic traits in healthy pet-owning household members. Underestimating normal household contact?. Comp. Immunol. Microbiol. Infect. Dis..

[19] Benito D., Gómez P., Aspiroz C., Zarazaga M., Lozano C., Torres C. (2015). Molecular characterization of *Staphylococcus aureus* isolated from humans related to a livestock farm in Spain, with detection of MRSA-CC130 carrying *mecC* gene: A zoonotic case?. Enferm. Infecc. Microbiol. Clin..

[20] Velasco V., Buyukcangaz E., Sherwood J.S., Stepan R.M., Koslofsky R.J., Logue C.M. (2015). Characterization of *Staphylococcus aureus* from humans and a comparison with isolates of animal origin, in North Dakota, United States. PLoS ONE.

[21] Sasaki T., Tsubakishita S., Tanaka Y., Sakusabe A., Ohtsuka M., Hirotaki S., Kawakami T., Fukata T., Hiramatsu K. (2010). Multiplex-PCR method for species identification of coagulase-positive staphylococci. J. Clin. Microbiol..

[22] Wayne P.A. (2018). Performance Standards for Antimicrobial Susceptibility Testing.

[23] Kehrenberg C., Schwarz S. (2006). Distribution of florfenicol resistance genes *fexA* and *cfr* among chloramphenicol-resistant *Staphylococcus* isolates. Antimicrob. Agents Chemother..

[24] Liu H., Wang Y., Wu C., Schwarz S., Shen Z., Jeon B., Ding S., Zhang Q., Shen J. (2012). A novel phenicol exporter gene, *fexB*, found in enterococci of animal origin. J. Antimicrob. Chemother..

[25] Schnellmann C., Gerber V., Rossano A., Jaquier V., Panchaud Y., Doherr M.G., Thomann A., Straub R., Perreten V. (2006). Presence of new *mecA* and *mph*(C) variants conferring antibiotic resistance in *Staphylococcus* spp. isolated from the skin of horses before and after clinic admission. J. Clin. Microbiol..

[26] Benito D., Lozano C., Rezusta A., Ferrer I., Vasquez M.A., Ceballos S., Zarazaga M., Revillo M.J., Torres C. (2014). Characterization of tetracycline and methicillin resistant *Staphylococcus aureus* strains in a Spanish hospital: Is livestock-contact a risk factor in infections caused by MRSA CC398?. Int. J. Med. Microbiol..

[27] Hauschild T., Vuković D., Dakić I., Ježek P., Djukić S., Dimitrijević V., Stepanović S., Schwarz S. (2007). Aminoglycoside resistance in members of the *Staphylococcus sciuri* Group. Microb. Drug Resist..

[28] Shopsin B., Gomez M., Montgomery S.O., Smith D.H., Waddington M., Dodge D.E., Bost D.A., Riehman M., Naidich S., Kreiswirth B.N. (1999). Evaluation of protein A gene polymorphic region DNA sequencing for typing of *Staphylococcus aureus* strains. J. Clin. Microbiol..

[29] Shopsin B., Mathema B., Alcabes P., Said-Salim B., Lina G., Matsuka A., Martinez J., Kreiswirth B.N. (2003). Prevalence of *agr* specificity groups among *Staphylococcus* aureus strains colonizing children and their guardians. J. Clin. Microbiol..

[30] Duran N., Ozer B., Duran G.G., Onlen Y., Demir C. (2012). Antibiotic resistance genes & susceptibility patterns in staphylococci. Indian J. Med. Res..

[31] Van Wamel W.J.B., Rooijakkers S.H.M., Van Kessel K.P.M., Van Strijp J.A.G., Ruyken M. (2006). The innate immune modulators Staphylococcal complement inhibitor and chemotaxis inhibitory protein of *Staphylococcus aureus* are located on β-Hemolysin-converting bacteriophages. J. Bacteriol..

[32] Kaspar U., von Lützau K., Schlattmann A., Rösler U., Köck R., Becker K. (2019). Zoonotic multidrug-resistant microorganisms among non-hospitalized horses from Germany. One Health.

[33] Islam M.Z., Espinosa-Gongora C., Damborg P., Sieber R.N., Munk R., Husted L., Moodley A., Skov R., Larsen J., Guardabassi L. (2017). Horses in Denmark are a reservoir of diverse clones of methicillin-resistant and -susceptible *Staphylococcus aureus*. Front. Microbiol..

[34] Parisi A., Caruso M., Normanno G., Latorre L., Miccolupo A., Fraccalvieri R., Intini F., Manginelli T., Santagada G. (2017). High Occurrence of Methicillin-Resistant *Staphylococcus aureus* in horses at slaughterhouses compared with those for recreational activities: A professional and food safety concern?. Foodborne Pathog. Dis..

[35] Agabou A., Ouchenane Z., Essebe C.N., Khemissi S., Tedj M., Chehboub E., Chehboub I.B., Sotto A., Dunyach-remy C., Lavigne J. (2017). Emergence of nasal carriage of ST80 and ST152 PVL+ *Staphylococcus aureus* isolates from livestock in Algeria. Toxins (Basel).

[36] Donker G.A., Deurenberg R.H., Driessen C., Sebastian S., Nys S., Stobberingh E.E. (2009). The population structure of *Staphylococcus aureus* among general practice patients from The Netherlands. Clin. Microbiol. Infect..

[37] Ben Said M., Abbassi M.S., Gómez P., Ruiz-Ripa L., Sghaier S., El Fekih O., Hassen A., Torres C. (2017). Genetic characterization of *Staphylococcus aureus* isolated from nasal samples of healthy ewes in Tunisia. High prevalence of CC130 and CC522 lineages. Comp. Immunol. Microbiol. Infect. Dis..

[38] Prestinaci F., Pezzotti P., Pantosti A. (2015). Antimicrobial resistance: A global multifaceted phenomenon. Pathog. Glob. Health.

[39] Guardabassi L., Schmidt K.R., Petersen T.S., Espinosa-Gongora C., Moodley A., Agersø Y., Olsen J.E. (2012). Mustelidae are natural hosts of *Staphylococcus delphini* group A. Vet. Microbiol..

[40] Ruiz-Ripa L., Gómez P., Alonso C.A., Camacho M.C., de la Puente J., Fernández-Fernández R., Ramiro Y., Quevedo M.A., Blanco J.M., Zarazaga M. (2019). Detection of MRSA of lineages CC130-mecC and CC398-mecA and *Staphylococcus delphini*-*lnu*(A) in magpies and cinereous Vultures in Spain. Microb. Ecol..

[41] Gharsa H., Slama K.B., Gómez-Sanz E., Gómez P., Klibi N., Zarazaga M., Boudabous A., Torres C. (2015). Characterisation of nasal *Staphylococcus delphini* and *Staphylococcus pseudintermedius* isolates from healthy donkeys in Tunisia. Equine Vet. J..

[42] Busscher J.F., Van Duijkeren E., Sloet Van Oldruitenborgh-Oosterbaan M.M. (2006). The prevalence of methicillin-resistant staphylococci in healthy horses in the Netherlands. Vet. Microbiol..

[43] Kern A., Perreten V. (2013). Clinical and molecular features of methicillin-resistant, coagulase-negative staphylococci of pets and horses. J. Antimicrob. Chemother..

[44] Sakr A., Brégeon F., Mège J.L., Rolain J.M., Blin O. (2018). Staphylococcus aureus nasal colonization: An update on mechanisms, epidemiology, risk factors, and subsequent infections. Front. Microbiol..

